# Integrated Analysis of ATAC-Seq and RNA-Seq Reveals the Signal Transduction Regulation of the Molting Cycle in the Muscle of Chinese Mitten Crab (*Eriocheir sinensis*)

**DOI:** 10.3390/biom16010108

**Published:** 2026-01-08

**Authors:** Zhen He, Jingjing Li, Jingjing Zhang, Ruiqi Zhang, Rongkang Tan, Jinsheng Sun, Bin Wang, Tong Hao

**Affiliations:** 1 College of Life Sciences, Tianjin Normal University, Tianjin 300387, China; zhenh2901@gmail.com (Z.H.); z18522131996@gmail.com (J.Z.); serenaalexande988@gmail.com (R.Z.); 2306712572qq@gmail.com (R.T.); skysjs@tjnu.edu.cn (J.S.); 2 Tianjin Key Laboratory of Animal and Plant Resistance, Tianjin Normal University, Tianjin 300387, China; 3 Tianjin Fisheries Research Institute, Tianjin 300384, China; jingjingli2511@gmail.com

**Keywords:** *Eriocheir sinensis*, molting cycle, signal transduction, GPCR, ATAC-seq, RNA-seq

## Abstract

Molting is a critical physiological process for the growth and development of *Eriocheir sinensis*. Any disruption in this process can significantly affect both survival rates and crab quality. The regulatory mechanisms of molting vary across different stages of the molting cycle and remain poorly understood. In this study, ATAC-seq and RNA-seq were combined to identify the integrated differentially expressed genes (IDEGs) in muscle across adjacent stages of the molting cycle. A total of 17, 491, 84, and 491 IDEGs were identified in the comparisons of inter-molt_vs_pre-molt, pre-molt_vs_molt, molt_vs_post-molt, and post-molt_vs_inter-molt stages, respectively. GO enrichment analysis of these IDEGs revealed several key signaling pathways involved in each adjacent molting stage. The GPCR signaling, steroid hormone-mediated signaling, and smoothened signaling pathways were all active across three molting transitions (pre-molt_vs_molt, molt_vs_post-molt, and post-molt_vs_inter-molt). Among them, the GPCR pathway played a dominant role throughout the process. Further structural analysis and RT-qPCR validation identified eight GPCRs involved in molting regulation: *GRM7* and *moody* were specific to the post-molt_vs_inter-molt stage; *Kpna6*, *ADRB2*, and *SSTR2* were unique to the pre-molt_vs_molt stage; *FMRFaR* and *gpr161* functioned in both post-molt_vs_inter-molt and pre-molt_vs_molt stages; and *mth2* was active in both post-molt_vs_inter-molt and molt_vs_post-molt stages. These findings improve the understanding of molting regulation and provide potential targets for further genetic improvement in *E. sinensis*.

## 1. Introduction

Molting is a pivotal physiological process governing growth and development in crustaceans, directly influencing key biological functions such as individual growth, tissue remodeling, and energy metabolism [1,2]. The Chinese mitten crab (*Eriocheir sinensis*), a commercially vital aquaculture species, undergoes approximately 18 molts throughout its life cycle, each associated with substantial increases in body weight and morphological transformations. However, aberrant molting frequently leads to elevated mortality or precocious maturation, posing significant constraints on aquaculture productivity. Elucidating the molecular mechanisms underlying molting regulation is therefore critical for optimizing breeding strategies and enhancing crab strain quality.

The crustacean molting cycle is divided into four distinct periods: inter-molt (period C), pre-molt (period D, including sub-periods D0, D1, and D3–4), molt (period E), and post-molt (periods A–B) [3]. Each stage is characterized by unique physiological and molecular signatures, with the antagonistic interaction between ecdysone and molt-inhibiting hormone (MIH) serving as the core regulatory mechanism to initiate cascades of molecular reactions. Muscle exhibits marked discontinuous growth during the molting cycle. Significant variations in muscle fiber diameter and density have been observed across different molting stages, with pronounced atrophy occurring during pre-molt and restoration post-molt [4]. Transcriptomic analyses have revealed stage-specific gene expression patterns in muscle: genes involved in muscle contraction, energy metabolism, and cytoskeletal remodeling are highly expressed during molting, whereas G protein-coupled receptors (GPCRs) and certain other signaling molecules show significant up-regulation in the post-molt period, highlighting the critical role of signaling pathways in dynamic muscle regulation [5,6].

Existing research has identified some molting-related genes in *E. sinensis*. For example, the ecdysone receptor gene *Ers-EcR* [7], the retinoid X receptor gene *Ers-RXR* [8], the chitinase gene *Ers-HXchit* [9], and the myostatin gene *Ers-MSTN* [10]. In terms of signaling pathways, the mTOR signaling pathway has been found to potentially participate in the molting process of the *E. sinensis* [11]. Additionally, a large number of molting-related genes have been identified through high-throughput sequencing. RNA-seq analysis of the hepatopancreas observed that differentially expressed genes (DEGs) enriched in the post-molt period were associated with energy consumption, while genes enriched in the inter-molt period were related to carbohydrate and lipid metabolism, as well as biosynthetic processes. In the pre-molt period, highly expressed genes were enriched in responses to steroid hormone stimulation and immune system development [12]. RNA-seq analysis of the claw muscle indicated that DEGs across different molting stages were primarily enriched in gene expression, protein synthesis, muscle development, new epidermis reconstruction, redox processes, and glycolysis [13]. RNA-seq analysis of the gills during four molting stages detected thousands of genes with altered expression, mainly involved in chitin degradation, exoskeleton reconstruction, and signaling processes. Up-regulated genes were enriched in several lipid-related metabolic pathways, as well as the three signaling pathways: “phosphatidylinositol signaling system,” “calcium signaling pathway,” and “GnRH signaling pathway” [14].

However, traditional methods have primarily focused on the study of individual genes, hindering a comprehensive dissection of the multi-gene regulatory networks. In addition, high-throughput studies have relied on RNA-seq alone, which carries limitations such as potential false positives and an incomplete representation of regulatory dynamics. The advent of Assay for Transposase-Accessible Chromatin using sequencing (ATAC-seq) has provided a novel framework for investigating chromatin accessibility and gene expression regulation [15]. Integrating ATAC-seq and RNA-seq data can minimize false positives and enable precise identification of DEGs and their cis-regulatory elements [16]. Sun et al. [5] integrated the data of two non-adjacent molting periods (pre-molt and post-molt) in muscle of *E. sinensis* to demonstrate that the GPCR signaling pathway dominates muscle signal transduction during the stages from pre-molt to post-molt periods. Wang et al. [6] further characterized stage-specific expression of GPCR family genes (e.g., *GRM7*, *mth2*) in the post-molt vs. inter-molt stages. However, while these studies both focused on the comparison of two periods in the molting process, a thorough multi-omics analysis spanning the entire molting cycle remains unexecuted, particularly in identifying conserved regulatory genes and core signaling pathways across sequential stages. Therefore, a comprehensive study that covers all four adjacent molting stages (C–D, D–E, E–A, A–C) is necessary to construct a complete regulatory landscape for molting process.

In this work, we focus on the investigation of the gene expressions in all four adjacent molting stages (C–D, D–E, E–A, A–C) in muscle of *E. sinensis* using integrated ATAC-seq and RNA-seq analyses. The aim of this study is to identify the stage-specific signaling pathways and genes across the entire molting process. The new ATAC-seq and RNA-seq data for the molt period (E) missing in previous studies are now provided. We hypothesize that integrated changes in chromatin accessibility and gene expression will reveal conserved signaling pathways, especially GPCR-mediated signaling, that coordinate muscle remodeling across the molting cycle. The candidate GPCRs were clarified via structural analysis and real-time quantitative PCR (RT-qPCR) validation. These findings can provide useful information for in-depth analysis of the continuous regulatory mechanism of the entire molting cycle of *E. sinensis* and ultimately benefit development of an anti-precocity strategy and cultivation of superior germplasm.

## 2. Materials and Methods

### 2.1. Animals and Sampling

#### 2.1.1. Experimental Animals and Culture Environment

Healthy juvenile *E. sinensis* crabs were obtained from Tianjin Xieyuan Aquaculture Co., Ltd. (Ninghe District, Tianjin, China). The average body length, body width, and weight of the crabs were 24.28 ± 1.54 mm, 26.97 ± 1.65 mm, and 8.41 ± 1.47 g, respectively. Prior to the experiment, the juvenile crabs were acclimatized in a plastic incubator (70 × 40 × 50 cm) for more than 7 days under controlled conditions, with a water temperature of 20 ± 1 °C and a natural light cycle. Compound feed was administered twice daily, with the feeding amount approximately 5% of the total body weight of the crabs. The siphon method was employed to remove residual feed and introduce fresh water, ensuring that the daily water exchange rate exceeded 50% to maintain water quality.

#### 2.1.2. Muscle Sample Collection During the Molt Period (Period E)

Juvenile male crabs in the molt period (period E) were selected as the research subjects. Macroscopically, the crab exoskeleton exhibited a translucent state, and a distinct separation interface was visible between the old and new exoskeletons [2]. A remarkable feature of the molting period is that the cracks between the cephalothorax and the abdominal segments keep increasing [2]. After being anesthetized by placing them on an ice plate, the sample crabs were rapidly dissected to obtain the muscle tissues from their walking legs. These tissues were immediately immersed in liquid nitrogen for cryopreservation and subsequently transferred to an ultra-low-temperature refrigerator at −80 °C for long-term storage. Muscle samples from crabs in different molting periods were collected. Three biological replicates were collected per period, with each sample obtained from one crab. Each sample was then divided into two parts, which were used for ATAC-seq and RNA-seq analyses, respectively. Therefore, in total, 24 samples from 12 crabs were obtained for ATAC-seq and RNA-seq analysis in four molting periods, of which 6 samples from 3 crabs in the molt period were used in this work. The other 18 samples from 9 crabs in pre-molt, post-molt and inter-molt periods were used for the ATAC-seq and RNA-seq analysis illustrated in our previously published work [5,6]. The cell viability of the samples was detected and counted using the Trypan blue staining method to ensure the reliability of subsequent molecular biology experiments.

### 2.2. Sequencing

#### 2.2.1. ATAC-Seq Sequencing

A total of three muscle samples, labeled as Mu_E_1, Mu_E_2, and Mu_E_3, were used for ATAC-seq to explore the characteristics of chromatin accessibility in muscle during the molt period. The ATAC-seq was carried out following the method described by Corces et al. [17]. Nuclei were extracted from the muscle samples, and the obtained nuclear pellet was re-suspended in the Tn5 transposase reaction mix from the Nextera DNA Flex Library Prep Kit (Illumina, San Diego, CA, USA). Tn5 transposase can efficiently cleave DNA in open chromatin regions and insert sequencing adapters into the cleavage sites simultaneously, enabling rapid enrichment of chromatin-accessible regions and library construction. This is a core step of the ATAC-seq technology, which can accurately capture the dynamic changes in chromatin accessibility, providing a key basis for subsequent differential accessibility analysis. Equimolar adapters 1 and 2 were added, followed by PCR amplification to construct the library. The library was subsequently purified with AMPure beads (Beckmann-Coulter, Pasadena, CA, USA), and the library quality was accurately evaluated using a Qubit (ThermoFisher, Waltham, MA, USA). The clustering of the index-coded samples was performed on a cBot Cluster Generation System (Illumina, San Diego, CA, USA) using TruSeq PE Cluster Kit v3-cBot-HS (Illumina, San Diego, CA, USA). The library preparations were sequenced on an Illumina NovaSeq platform (Illumina, San Diego, CA, USA) at Novogene Co., Ltd. (Chaoyang District, Beijing, China), generating 150 bp paired-end reads.

#### 2.2.2. RNA-Seq Sequencing

Total RNA was extracted from three molt-period muscle samples (Mu_E_1, Mu_E_2, and Mu_E_3) of *E. sinensis* using the TRIzol reagent (Invitrogen, Carlsbad, CA, USA), following the manufacturer’s protocol. RNA integrity and concentration were assessed using the RNA Nano 6000 Assay Kit (Agilent Technologies, Santa Clara, CA, USA) on an Agilent Bioanalyzer 2100 system (Agilent Technologies, Santa Clara, CA, USA) to ensure the sample quality for downstream sequencing. Library preparation was performed using the NEBNext^®^ Ultra™ RNA Library Prep Kit for Illumina^®^ (NEB, Ipswich, MA, USA). The cDNA libraries were amplified via PCR, and library quality (size distribution and molarity) was validated on the Agilent Bioanalyzer 2100 system (Agilent Technologies, Santa Clara, CA, USA). Sequencing was conducted on an Illumina NovaSeq 6000 platform (Illumina, San Diego, CA, USA) at Novogene Co., Ltd. (Chaoyang District, Beijing, China), generating 150 bp paired-end reads.

### 2.3. Data Analysis

#### 2.3.1. ATAC-Seq Data Analysis

After sequencing, a series of data processing and analysis was carried out. The Nextera adaptor sequences in the reads were trimmed using skewer (version 0.2.2, Chinese Academy of Inspection and Quarantine, Beijing, China) [18]. Reads meeting any of the following criteria were removed from the raw data to obtain clean data: reads with over 40% of bases having a quality score below 15, reads containing more than 6 ‘N’ bases, reads with adapter sequences, and reads shorter than 18 bp after trimming. The Q20 and Q30 values of the raw data and clean data were calculated. The reads were aligned to the reference genome downloaded from the NCBI BioProject database (accession number PRJNA555707) using BWA with standard parameters [19]. The same version of reference genome was used in this work and our previously published ATAC-seq datasets (GSE206233, GSE281094) [5,6]. Subsequently, high-quality (MAPQ ≥ 13), non-mitochondrial chromosomes and properly paired reads (longer than 18 nt) were further filtered. The read signals in the upstream and downstream 3 kb region of transcription start sites (TSS) and structural genes were statistically analyzed with deepTools software (version 3.0.2, Max Planck Institute of Immunobiology and Epigenetics, Freiburg, Germany) [20]. All peak calling was performed per sample with MACS2 (version 2.1.2, Dana-Farber Cancer Institute and Harvard School of Public Health, Boston, MA, USA) using “MACS2 call-peak—nomodel—keepdup all—call-summits” [21].

The distribution of peaks across various functional regions was statistically analyzed with ChIPseeker software (version 1.38.0, Jinan University, Guangzhou, China) [22]. The mapping relationship between peaks and functional regions follows a priority order of promoter-TSS, 5′UTR, 3′UTR, exon, intron, downstream, and distal intergenic. That is, if a peak is located at a position that could be an exon of one gene and simultaneously an intron of another gene, according to the above priority order, it will be considered annotated to the exon rather than the intron.

Combined with the previous ATAC-seq sequencing data of *E. sinensis* during the inter-molt period (period C), pre-molt period (period D), and post-molt period (period A) (GEO database with accession numbers GSE206233 and GSE281094) [5,6], differential analysis of each adjacent stage (C vs. D, D vs. E, E vs. A, A vs. E) was performed using the DESeq2 R package (version 1.20.0, Harvard School of Public Health, Boston, MA, USA) [23]. The average FoldEnrich values of peaks in the biological replicates were used when comparing different molting periods. Peaks with *p*-value ≤ 0.05 and |log_2_FoldEnrich| ≥1 were defined as the differentially enriched peaks (DEP).

#### 2.3.2. RNA-Seq Data Analysis

Raw sequencing data were processed to remove low-quality reads, adapter-contaminated sequences, and reads with ≥10% undetermined bases (poly-N) using Trimmomatic (version 0.39, Max Planck Institute of Molecular Plant Physiology, Golm, Germany) [24]. Quality metrics of the resulting clean reads, including Q20, Q30 and GC content, were calculated using FastQC (version 0.11.9, Babraham Institute, Cambridge, UK) [25] The *E. sinensis* reference genome (accession number PRJNA555707, obtained from NCBI BioProject database) was indexed with Hisat2 (version 2.0.5, Johns Hopkins University, Baltimore, MD, USA) [26], and clean reads were aligned to the reference genome using the same tool with default parameters. The same version of reference genome was used in this work and our previously published RNA-seq datasets (GSE206233, GSE281094) [5,6]. Based on the position information of gene alignment on the reference genome, the number of reads covered by each gene from start to end is counted. Parts of the reads were filtered out, including the reads with comparison quality values below 10, reads on non-comparative pairs, and reads aligned to multiple regions of the genome. The FeatureCounts (version 1.5.0-p3, The University of Melbourne, Parkville, VIC, Australia) [27] were used for the reads filter. Gene expression levels were quantified by calculating fragments per kilobase of exon model per million mapped fragments (FPKM) using FeatureCounts (version 1.5.0-p3, The University of Melbourne, Parkville, VIC, Australia) [27], which counts reads uniquely mapped to each gene. The gene expression levels for each sample were quantitatively analyzed separately.

Combined with the previous RNA-seq sequencing data of *E. sinensis* during the inter-molt, pre-molt, and post-molt periods (GEO database with accession numbers GSE206233 and GSE281094) [5,6], differential expression analysis of each adjacent stage (C vs. D, D vs. E, E vs. A, A vs. E) was performed using the DESeq2 R package (version 1.20.0, Harvard School of Public Health, Boston, MA, USA) [23]. Genes with adjusted *p*-value (padj) ≤ 0.05 (Benjamini–Hochberg correction) and |log_2_FoldChange| ≥ 1 were identified as the DEGs.

The ATAC-seq and RNA-seq datasets for Mu_E_1, Mu_E_2, and Mu_E_3 have been deposited in the Gene Expression Omnibus (GEO) database under accession number GSE299867, ensuring reproducibility and public accessibility of the results.

#### 2.3.3. Integrated Analysis

To identify the genes with concordant regulatory and expression patterns, the expression profiles of genes associated with DEPs from ATAC-seq were cross-referenced with DEGs from RNA-seq. The promoter in ATAC-seq analysis is defined as the ±2 kb region flanking each gene’s TSS, and the structural gene encompasses the full transcribed sequence from TSS to transcription termination site (TTS), including exons and introns. For DEP-gene mapping, a hierarchical approach was adopted: peaks overlapping the promoter region were first assigned to their corresponding genes; peaks not overlapping any promoter were mapped to the gene associated with the nearest TSS, regardless of distance. All DEGs from RNA-seq were first intersected with genes linked to DEPs from ATAC-seq. These intersected genes were then classified into up-regulated or down-regulated categories based on their expression trends in RNA-seq. Genes with both differential expression and differentially accessible peaks were identified as integrated differential expression genes (IDEGs). Goseq (version 1.0, The Walter and Eliza Hall Institute of Medical Research, Parkville, Australia) [28] was used for Gene Ontology (GO) annotations of the IDEGs. The GO annotations focused on the biological processes, cellular components, and molecular functions. It helps to identify the signaling pathways involved in different molting stages. 

### 2.4. Structural Analysis of Candidate GPCRs

To characterize the molecular architecture of candidate GPCR genes potentially involved in GPCR signaling transduction, a multi-layered structural prediction strategy was employed. Transmembrane domain topology was first analyzed using TMHMM 2.0 [29] (https://services.healthtech.dtu.dk/services/TMHMM-2.0/ (accessed on 5 June 2025)) to identify α-helical membrane-spanning regions. Secondary structure predictions were conducted using PSIPRED 4.0 [30] (https://predictprotein.org/ (accessed on 5 June 2025)) to confirm the presence of periodic α-helical structures within predicted transmembrane domains. For tertiary structure modeling, SWISS-MODEL [31] (https://swissmodel.expasy.org/ (accessed on 5 June 2025)) was utilized to generate the homology-based 3D structures to highlight the transmembrane helix bundle and extracellular ligand-binding domains for potential G-protein interaction sites. Proteins identified by at least two tools as having a hallmark 7-TMH structure were considered as GPCR proteins.

### 2.5. Gene Expression Verification

To validate the differential expression profiles of the GPCR genes identified through integrated ATAC-seq and RNA-seq analyses, RT-qPCR was performed on candidate genes. Crabs in the same batch as those used in ATAC-seq/RNA-seq analysis were collected for RT-qPCR validation. Three biological replicates per period were collected for pre-molt, molt, and post-molt periods, with each consisting of the pooled tissue from three crabs to minimize individual variation. In total, 18 tissue samples from 18 crabs were used, of which 9 samples were used for the validation of gene expressions in D vs. E stages, and the other 9 were used for E vs. A stages. Total RNA was extracted using TRIzol reagent (Invitrogen, San Diego, CA, USA), and cDNA synthesis was carried out with PrimeScript™ RT Master Mix (Takara, Shiga, Japan), employing β-actin as the endogenous control for normalization.

Primers were designed to target conserved coding regions, with amplicon sizes optimized for efficiency (100–200 bp). The primer sequences and efficiencies are shown in Supplementary Table S1. The primer efficiency ranged between 90.72% and 109.53%. The 20-μL reaction mixture included 1 μL of cDNA, 0.4 μM of each primer, and TB Green^®^ Premix Ex Taq™ II (Takara, Japan), amplified on an ABI QuantStudio 5 system under the following conditions: 30 s at 95 °C for initial denaturation, followed by 40 cycles of 5 s at 95 °C and 30 s at 60 °C, with a final melt curve analysis to confirm product specificity. Relative expression levels were calculated using the 2^−ΔΔCt^ method; statistical significance was determined via two-tailed Student’s *t*-tests (*p* ≤ 0.05) with Excel formula functions.

## 3. Results

### 3.1. ATAC-Seq Analysis of Chromatin Accessibility in Different Molting Stages

The ATAC-seq analysis of *E. sinensis* muscle in the molt period uncovered distinctive chromatin accessibility landscapes during this biological process. The results and quality control parameters for ATAC-seq of the three samples in the molt period are shown in Table 1. The average Q20 and Q30 for the clean reads across the three samples were 94.4% and 87.5%, respectively, indicating high sample quality. The average alignment rate of the clean reads to the reference genome was 75.1%. The number of detected peaks ranged from 44,610 to 52,473, with summit counts ranging from 56,607 to 68,956.

**Figure 1:**
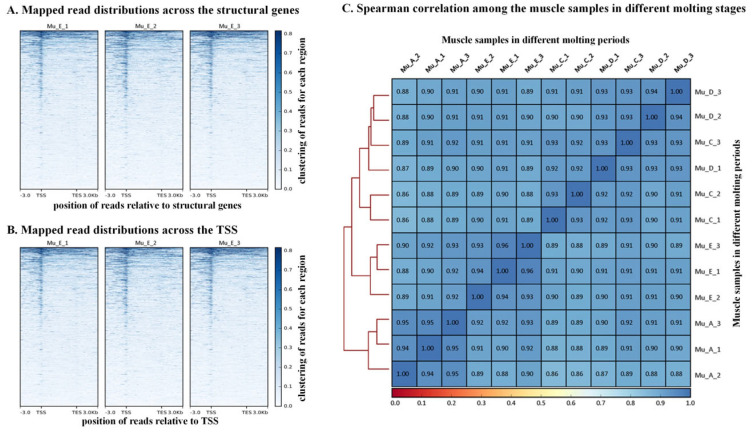
Quality control results of the ATAC-seq analysis. “A” represents the post-molt period, “C” stands for the inter-molt period, “D” refers to the pre-molt period, and “E” indicates the molt period.

The enriched reads at the structural genes (Figure 1A) suggest that many genes were active and played functional roles in the molt period. In addition, pronounced enrichment of reads was detected at TSS (Figure 1B), which are key regulatory nodes within promoter regions. This indicates active transcription during the molt period. The distribution of peaks in gene functional regions was further analyzed. The percentages of peaks enriched in the functional regions are as follows: Promoter-TSS 26.4%, 5′UTR 0.04%, 3′UTR 0.2%, exon 1.7%, intron 19.1%, downstream 1.4%, and distal intergenic 51.3%. The distal intergenic and Promoter-TSS regions exhibited the highest accessibility, highlighting their importance in the transcriptional process. The high accessibility of distal intergenic and Promoter-TSS regions featured many distal regulatory elements that may interact with promoters to modulate gene networks. The structural genes, including introns and exons, show a little lower overall accessibility compared to promoters. This indicates that the expression of certain genes may have only regulatory functions instead of encoding proteins with actual functions. Collectively, these findings highlight the collaborative roles of structural genes, promoter-TSS regions, and intergenic regulatory elements in coordinating molting-associated gene expression.

Spearman correlation analysis of read counts (Figure 1C) in all four molt periods was performed. The result showed robust intra-group consistency, with correlations exceeding 0.90 among replicate samples within the same molting period. This validates the reliability of sample quality and the reproducibility of chromatin accessibility profiles, providing a robust foundation for downstream mechanistic investigations.

**Figure 2:**
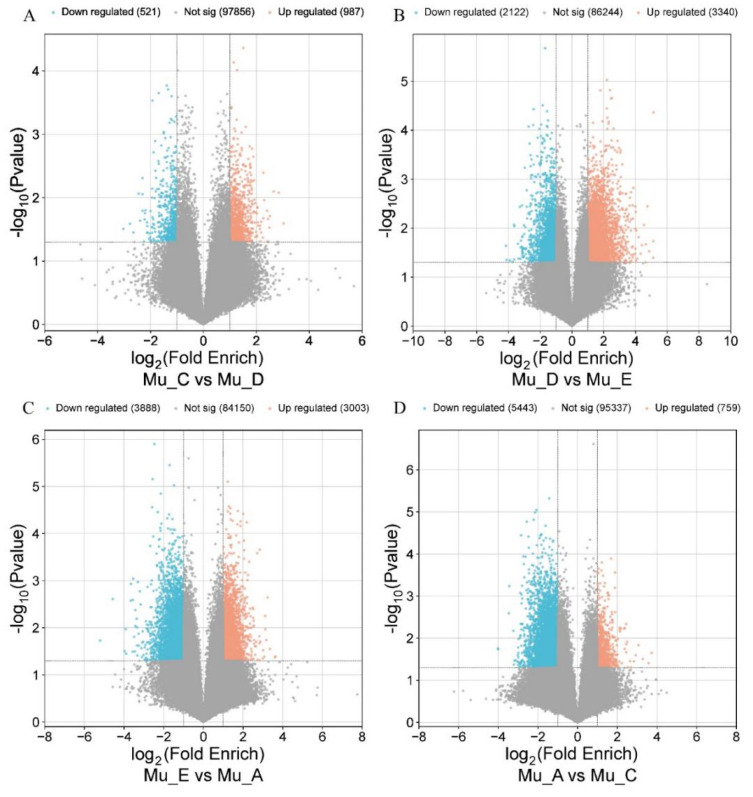
DEPs identified by ATAC-seq in *E. sinensis* muscle across different molting stages. (**A**) Comparison between inter-molt and pre-molt periods; (**B**) Comparison between pre-molt and molt periods; (**C**) Comparison between molt and post-molt periods; (**D**) Comparison between post-molt and inter-molt periods.

Combined with the ATAC-seq data of other molting periods, the DEPs showing regulatory changes of *E. sinensis* muscle across various molting stages are visualized with volcano plots (Figure 2). The ATAC-seq analysis revealed marked dynamic variations in chromatin accessibility across molting stages in *E. sinensis* muscle, and the numbers of DEPs reflect distinct regulatory demands. The transition from the molt period (Mu_E) to the post-molt period (Mu_A) exhibited the highest number of DEPs (6891), likely attributed to extensive transcriptional reprogramming required for muscle recovery from mechanical stress during molting and coordinated exoskeleton hardening. Intermediate DEP counts were observed in the Mu_A vs. Mu_C (6202) and Mu_D vs. Mu_E (5462) stages: the former reflects the down-regulation of molt-specific regulatory programs and reactivation of inter-molt homeostatic networks, while the latter corresponds to the transcriptional coordination of exoskeleton degradation, new cuticle formation, and muscle contraction necessary for molting. In contrast, the Mu_C vs. Mu_D stages yielded the fewest DEPs (1508), consistent with the gradual initiation of early molt preparation involving only modest activation of ecdysteroid-responsive genes without large-scale network rewiring. These findings highlight that the magnitude of chromatin accessibility changes is tightly coupled with the physiological complexity of each molting stage, with the most pronounced regulatory shifts occurring during post-molt tissue repair and exoskeleton maturation and minimal changes during the pre-molt priming phase when homeostasis is still largely maintained.

### 3.2. RNA-Seq Analysis of Gene Expression in Different Molting Stages

The RNA-seq sequencing results and quality-control metrics for the three samples in the molt period are presented in Table 2. The average clean ratio of the three samples is 96.7%. The mean Q20 and Q30 values across all samples were 96.3% and 91.5%, respectively, demonstrating the high quality of the sequencing libraries. The average mapping efficiency to the reference genome across the three samples was 82.5%.

**Figure 3:**
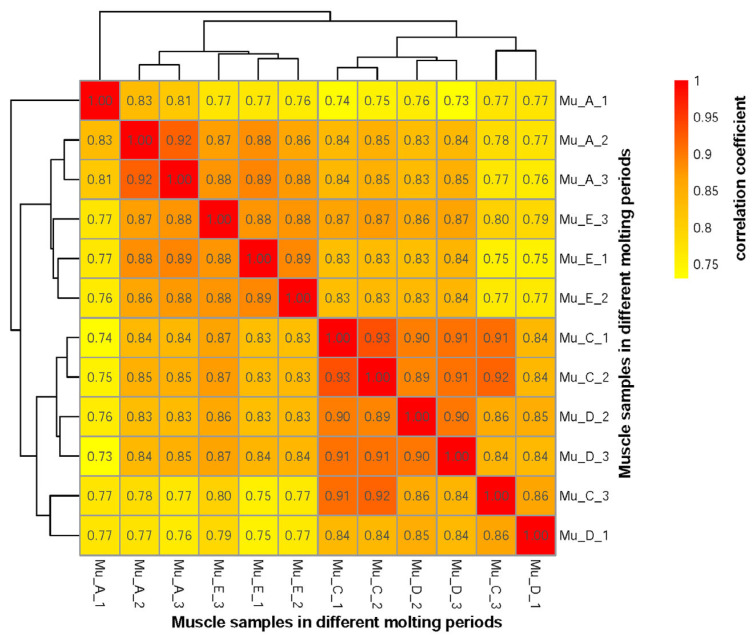
Spearman correlation heatmap of samples from *E. sinensis* muscle across different molting periods. Mu_A: sample in the post-molt period of muscle. Mu_C: sample in the inter-molt period of muscle. Mu_D: sample in the pre-molt period of muscle. Mu_E: sample in the molt period of muscle.

The robust correlations in gene expression profiles of *E. sinensis* muscle across different molting stages are depicted in the Spearman correlation heatmap (Figure 3). Samples from the post-molt period (Mu_A_1, Mu_A_2, Mu_A_3), molt period (Mu_E_1, Mu_E_2, Mu_E_3), inter-molt period (Mu_C_1, Mu_C_2, Mu_C_3), and pre-molt period (Mu_D_1, Mu_D_2, Mu_D_3) exhibited distinct clustering patterns, with intragroup correlations ranging from 0.8137 to 0.9342. Specifically, inter-molt samples showed strong internal consistency (0.9107–0.9342), mirrored by similarly high values in post-molt (0.8137–0.9209), pre-molt (0.8353–0.9013) and molt (0.8766–0.8878) groups, which underscores the technical reproducibility and biological consistency. As Spearman correlation coefficients over 0.8 are considered as very strong correlations [32], these results validate the reliability of RNA-seq data and emphasize the stage-specific gene expression patterns critical for molting-associated physiological transitions. These results provide a robust foundation for identifying DEGs and their roles in molting regulation.

**Figure 4:**
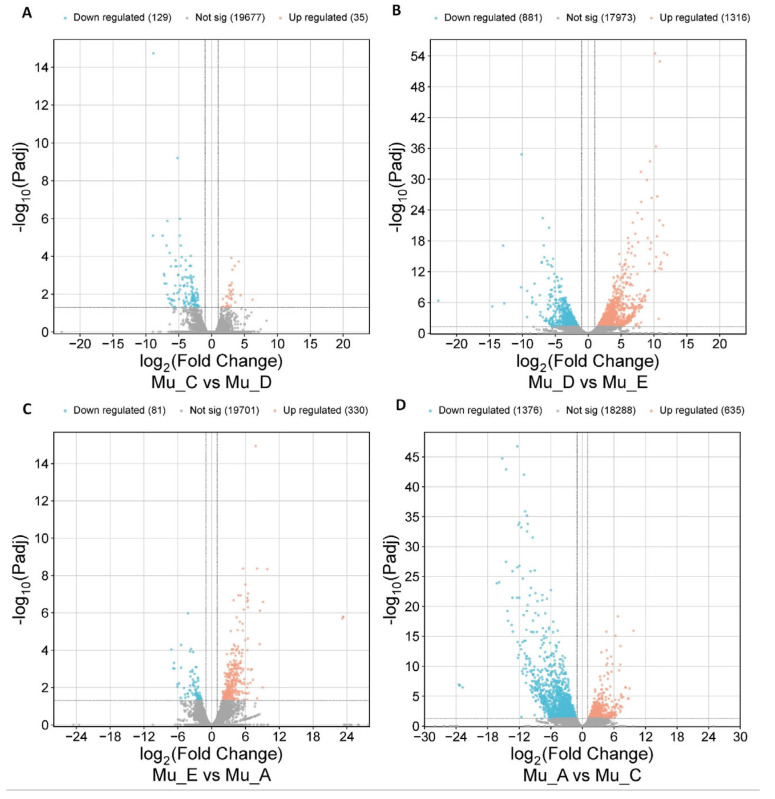
The DEGs from RNA-seq in *E. sinensis* muscle across different molting stages. (**A**) Comparison between inter-molt and pre-molt periods; (**B**) Comparison between pre-molt and molt periods; (**C**) Comparison between molt and post-molt periods; (**D**) Comparison between post-molt and inter-molt periods.

Integrated with the RNA-seq data from other molting stages (GSE206233 and GSE281094) [5,6], the DEGs of all the adjacent molting stages were identified (Figure 4). Mu_D vs. Mu_E and Mu_A vs. Mu_C stages exhibited the highest number of DEGs, with 2197 and 2011, respectively, indicating a dramatic transcriptional rewiring likely required for the execution of the molt and the transition from post-molt to inter-molt stages. The Mu_E vs. Mu_A transition presented an intermediate number of DEGs (411) associated with post-molt recovery. However, in the results of ATAC-seq, this stage contained the highest number of DEPs, indicating that although both chromatin accessibility and mRNA expression are related to transcriptional processes, they are not completely identical. The mutual corroboration of the two techniques can further enhance the credibility of the data. Mu_C vs. Mu_D transition showed the fewest DEGs (164), suggesting a preparatory phase with subtle transcriptional priming, which agrees with the results of ATAC-seq.

### 3.3. Integration of ATAC-Seq and RNA-Seq for IDEG Analysis

**Figure 5:**
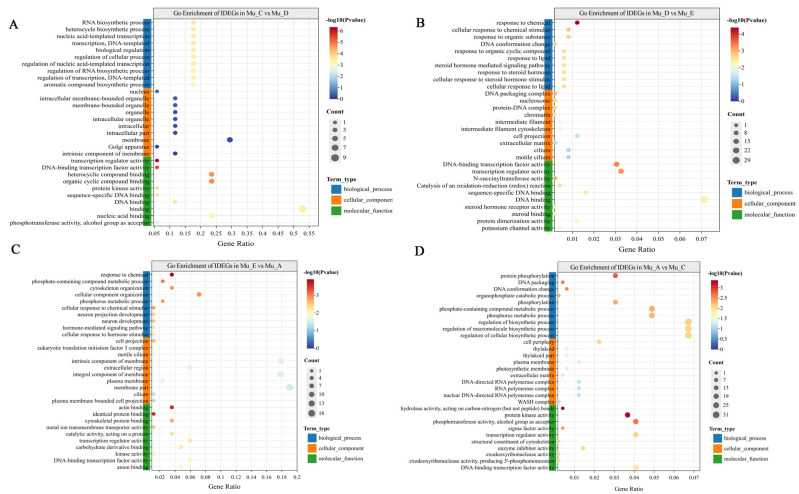
GO enrichment analysis of IDEGs across four molting stages in E. sinensis muscle. (**A**): Mu_C vs. Mu_D stages, (**B**): Mu_D vs. Mu_E stages, (**C**): Mu_E vs. Mu_A stages, (**D**): Mu_A vs. Mu_C stages.

Through the integrated analysis of ATAC-seq and RNA-seq data, IDEGs exhibiting both differential expression and chromatin accessibility were identified across the four molting stages in *E. sinensis* muscle (Table 3, Supplementary Table S2). The GO enrichment was further analyzed for these IDEGs. During the transition from inter-molt to pre-molt periods (Mu_C vs. Mu_D, Figure 5A), 17 genes showed significant expression changes. These genes were primarily associated with fundamental cellular processes such as biosynthesis of RNA, heterocycle and aromatic compound synthesis processes, DNA-templated transcription and regulation of these biological processes. This might be associated with the physiological preparations for the impending molting process. Notably, the GO enrichment analysis revealed that “RNA biosynthetic process” was most significantly enriched, indicating that the synthesis of large amounts of RNA was required for producing proteins related to the transition from inter-molt to pre-molt periods. At the molecular function level, “transcription regulator activity” and “DNA-binding transcription factor activity” were significantly enriched. This underscores the critical role of transcription factors in precisely controlling the expression of molting-related genes. In addition, the most significant enrichment of “nucleus” at the cellular component level further proved the active transcription process during this stage.

In the pre-molt to molt stages (Mu_D vs. Mu_E, Figure 5B), 491 IDEGs were identified. The GO enrichment analysis displayed significant enrichment of “response to chemical”, “cellular response to chemical stimulus”, and “response to steroid hormone”. This indicates that the molting process is regulated by diverse chemical signals. The enrichment of “steroid hormone-mediated signaling pathway” emphasized the indispensable role of ecdysteroids in coordinating cellular behaviors and physiological activities through regulating gene expression. It may thereby ensure the smooth progression of molting. At the molecular function level, “steroid binding” and “steroid hormone receptor activity” were significantly enriched. This highlights the initial key step of hormone signal transduction through the binding of ecdysteroids to their respective receptors. This stage includes some typical sterol receptors, such as *gpr161*, which were enriched in “steroid hormone receptor activity” and “steroid hormone-mediated signaling pathway”. This was found in both mice and zebrafish to mediate the signaling regulation [33,34]. At the cellular component level, notable enrichments were found in “extracellular matrix” and “intermediate filament cytoskeleton”. The latter is critical for exoskeleton degradation, structural remodeling, and signal transduction during the mechanical stress of shell shedding and body expansion.

In molt to post-molt stages (Mu_E vs. Mu_A, Figure 5C), 84 IDEGs were enriched in metabolic processes, including “phosphate-containing compound metabolic process”, “phosphorus metabolic process”, and pathways related to cellular structure organization, such as “cytoskeleton organization” and “cellular component organization”. This underscores the importance of phosphorus metabolism and cytoskeletal assembly when crabs recover after molting. The GO enrichment analysis revealed significant enrichment of “response to chemical stimulus” and “cellular response to chemical stimulus”, suggesting that during this stage, the molting process is regulated by certain chemical signals. The enrichment of hormone-related items, including “hormone-mediated signaling pathway” and “cellular response to hormone stimulus”, indicated the central role of hormones in regulating the expression of molting-related genes. The *mth2* gene was enriched in the “hormone-mediated signaling pathway” in this stage. This has been found to affect the lifespan regulation in fruit flies (*D. melanogaster*) [35]. At the molecular function level, the enrichment of “transcription regulator activity” and “DNA-binding transcription factor activity” reflected the involvement of numerous gene expression changes during molting. The transcriptional regulators may control the molting process by binding to DNA and regulating the transcription of relevant genes.

The post-molt to inter-molt transition (Mu_A vs. Mu_C, Figure 5D) involved 491 IDEGs. The GO enrichment analysis revealed significant enrichments in GO categories closely related to protein phosphorylation and phosphoregulatory functions. These are vital for cellular signal transduction, protein activity modulation, and preparing for the subsequent molting cycle. In the biological process category, terms such as “protein phosphorylation” and “phosphorylation” were prominently enriched. This indicates that protein modification through phosphorylation is actively regulated during the post-molt phase. Phosphorylation serves as a key mechanism for signal transduction. It participates in energy-related metabolism and contributes to the reestablishment of cellular homeostasis. Regarding molecular function, “protein kinase activity” and “phosphotransferase activity, alcohol group as acceptor” were significantly enriched. Protein kinases can mediate the phosphorylation of target proteins, thereby regulating their activity and transmitting signals in molting-associated pathways. These phosphoregulatory functions emphasize the central role of phosphate-mediated modifications in coordinating cellular responses during the post- to inter-molt transition.

The GO enrichment analysis of these stage-specific IDEGs constructs a detailed molecular landscape. The pivotal role of signal transduction in regulating molting-related physiological adaptations is prominently revealed. Signaling-related pathways were almost enriched in all the molting stages. They were found to be possibly correlated with certain critical biological processes for molting, such as exoskeletal organization, cellular component organization and hormonal regulation. The GO enrichment analysis directly linked the transcriptional changes to the coordinated activation of molecular cascades essential for successful ecdysis. These findings highlight that the signaling pathways may act as a central hub in the regulatory networks throughout the molting process and coordinate the integration of diverse biological functions to ensure the seamless progression of the molting cycle in *E. sinensis*.

### 3.4. Signaling Pathway Analysis of IDEGs

**Figure 6:**
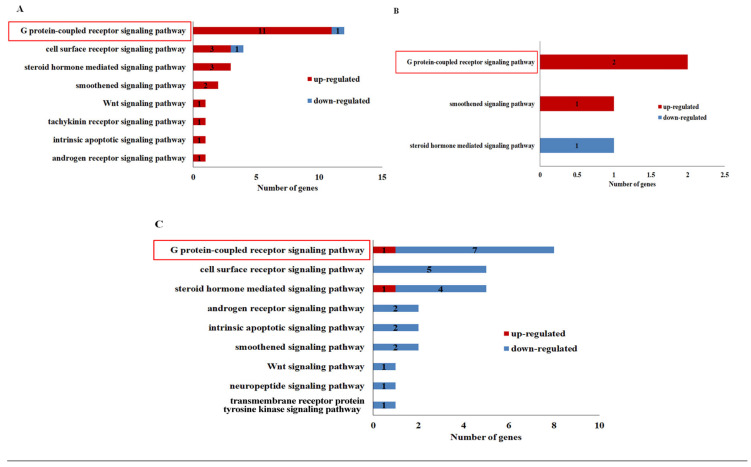
Enrichment analysis of signaling pathways across different molting stages. (**A**): Mu_D vs. Mu_E stages, (**B**): Mu_E vs. Mu_A stages, (**C**): Mu_A vs. Mu_C stages. The GO enrichment analysis was conducted with Goseq [28], and the bar chart output was generated with Excel. The GPCR signaling pathway dominating most of the molting stages is highlighted with red rectangle.

In order to further investigate the involvement of signaling transduction in the molting process, the signaling pathways in different molting stages were further analyzed based on the GO enrichment results, except for the Mu_C vs. Mu_D stages, where none of the signaling pathways were enriched. In the Mu_D vs. Mu_E stages (Figure 6A), the “G protein-coupled receptor signaling pathway” (GO:0007186) emerges as the most prominent. It encompasses the highest number of genes (12 genes), which is substantially more than other pathways, such as the cell surface receptor signaling pathway (GO:0007166, 4 genes) and the steroid hormone-mediated signaling pathway (GO:0043401, 3 genes). This dominance underscores the critical role of GPCR signaling in the transition from the pre-molt to molt periods. In the molt to post-molt stages (Mu_E vs. Mu_A, Figure 6B), the GPCR signaling pathway (GO:0007186) remains a key player, though the number of associated genes decreases to 2. Pathways like smoothened signaling (GO:0007224) and steroid hormone-mediated signaling pathway (GO:0043401) also come into play, each with one gene. This shift may reflect the changes in regulatory needs. In the process of Mu_A vs. Mu_C stages (Figure 6C), representing the transition from the post-molt to inter-molt period, the GPCR signaling pathway (GO:0007186) continues to be the most enriched, with 8 genes, followed by steroid hormone-mediated signaling (GO:0043401, 5 genes) and cell surface receptor signaling pathway (GO:0007166, 5 genes). The persistent enrichment of GPCR signaling across all comparisons highlights its fundamental importance in coordinating the physiological changes required for molting. In addition, the varying enrichment of other pathways, such as Wnt signaling (GO:0016055), tyrosine kinase signaling (GO:0007169), and intrinsic apoptosis signaling pathways (GO:0097193) in different stages further illustrates the stage-specific regulatory mechanisms adjusting the molting process in *E. sinensis*.

It is worth noting that none of the genes are active across all the molting stages, even for the same signaling pathway. This situation precisely illustrates the complexity of the molting process in *E. sinensis*. Even if the same signaling pathway is involved, the genes that are active during different molting stages differ.

### 3.5. Structural Analysis of Candidate GPCR Genes

Through the enrichment analysis of signaling pathways, the GPCR signaling pathway was found to play an important role in almost all molting stages. To characterize the specific GPCRs functioning in different molting stages, the structure of the proteins encoded by the genes in GPCR signaling pathways in different stages was analyzed using TMHMM, PSIPRED and SWISS-MODEL tools to assess the transmembrane helices, secondary structures, and tertiary conformations. The genes in the GPCR signaling pathways were first filtered with the GO function of “G protein-coupled receptor activity” (GO:0004930). Those annotated with GO:0004930 were further confirmed for the structure of associated proteins. The proteins were confirmed as GPCR family members with the confirmation of the canonical seven-transmembrane helical (7-TMH) structure by at least two prediction tools. The predicted structures of these proteins are shown in Supplementary Figures S1–S3.

In the Mu_A vs. Mu_C stages, 8 GPCR genes were initially enriched in GPCR signaling pathways, of which 5 genes (evm.TU.CM024127.1.244, evm.TU.CM024146.1.116, evm.TU.CM024164.1.176, evm.TU.CM024134.1.314, evm.TU.CM024100.1.415) exhibited GPCR activity and then underwent structural validation, while the other three genes (evm.TU.CM024099.1.306, evm.TU.CM024125.1.103, evm.TU.CM024097.1.36) have no GPCR activity according to their GO annotations. As detailed in Table 4, all 5 genes with GPCR activity displayed consistent 7-TMH features.

In the Mu_D vs. Mu_E stages, 12 GPCR genes were enriched, with 7 demonstrating GPCR activity. Structural analysis of these 7 genes confirmed 5 that harbored conserved 7-TMH structures, validating their GPCR membership. In these genes, evm.TU.CM024164.1.176 and evm.TU.CM024127.1.244 were also functioning in the Mu_A vs. Mu_C stages. Evm.TU.CM024165.1.210 (*ADRB2*) showed 8 TMHs based on the prediction of TMHMM, but actually the probability for one of its TMHs is lower than 0.5 (see Supplementary Figure S1). It might be an algorithm-driven over-prediction. TMHMM relies primarily on hydrophobicity profiles, and the short, highly hydrophobic N-terminal segment of crustacean *ADRB2* (with sequence divergence from mammalian templates) is misclassified as an extra transmembrane helix. It is a common limitation of this tool for non-mammalian GPCRs. In addition, both PSIPRED and SWISS-MODEL predicted 7 TMHs for this gene. Therefore, evm.TU.CM024165.1.210 was finally identified to have 7 TMHs, which conforms to the characteristic structure of GPCR.

In the Mu_E vs. Mu_A stages, two genes were found in GPCR signaling pathway, and both of them had GPCR activity. Only evm.TU.CM024134.1.314 showed 7-TMH conformation in all three prediction tools, which was also found in the Mu_A vs. Mu_C stages.

The conserved 7-TMH conformation of GPCRs is critical for transmembrane signal transduction. The consistency of predictions by TMHMM, PSIPRED, and SWISS-MODEL for the candidate genes further validates some of them as GPCR genes. The stage-specific expression patterns provide preliminary evidence for their roles in molting-associated signaling. These findings establish a structural basis for further functional investigations into how these receptors modulate downstream pathways during crustacean molting, contributing to mechanistic insights into discontinuous growth and developmental regulation.

### 3.6. RT-qPCR Verification of GPCR Genes

Some of the GPCR genes involved in different molting stages are duplicated. Excluding the duplicated genes, there are a total of 8 GPCR genes involved in the molting process. Based on the RNA-seq results, the expression of these genes at different molting stages is shown in Table 5. RT-qPCR was further performed for these genes to validate the sequencing results.

**Figure 7:**
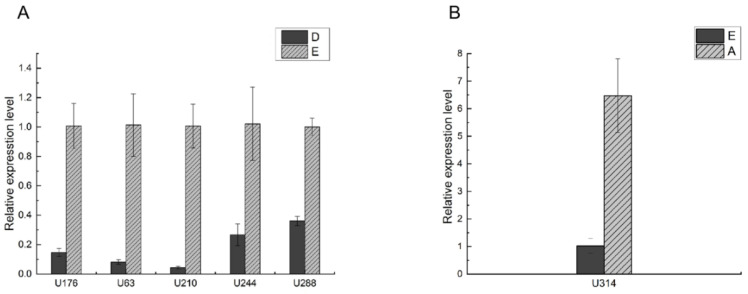
The results of RT-qPCR for the candidate GPCR genes. (**A**) Results for the candidate GPCR genes in the Mu_D vs. Mu_E stages; (**B**) Results for the candidate GPCR gene in the Mu_E vs. Mu_A stages. The abbreviation in this figure is cross-referenced with Table 5.

The expression of the five IDEGs in the Mu_A vs. Mu_C stages was verified in our previous work [6], which completely agrees with the results in this work. The samples used in this study were obtained from the same group of crabs as those in our previous paper [6], and all four adjacent transitions (C–D, D–E, E–A, A–C) were analyzed in a unified IDEG framework. Therefore, the data consistency between the two sets of works is ensured. Since these five genes have already been validated by RT-qPCR experiments in the earlier paper, to avoid redundant experimentation, this study does not repeat the validation for the expression of these five genes in the Mu_A vs. Mu_C stages. Therefore, there are five genes (U63, U210, U288, U176, U244) in the Mu_D vs. Mu_E stages and one gene, U314, in the Mu_E vs. Mu_A stages whose expression needs to be validated. The RT-qPCR results are shown in Figure 7. The expression profiles of these genes are consistent with the RNA-seq results.

## 4. Discussion

Based on the ATAC-seq and RNA-seq techniques, the IDEGs across four different molting stages were investigated. The highest number of IDEGs was found in the Mu_D vs. Mu_E and Mu_A vs. Mu_C stages, with both stages including 491 IDEGs. The number of up-regulated genes in the Mu_D vs. Mu_E stages was significantly higher than that of down-regulated genes, whereas the opposite is observed in the Mu_A vs. Mu_C stage. This indicates that a large number of genes are highly expressed to complete biological processes such as energy metabolism and hormone synthesis required for molting. After molting is completed, during the transition from the post-molt to the inter-molt stages, the highly expressed genes gradually return to normal expression levels, entering a long-term stable phase of metabolism and energy storage in preparation for the next molting cycle. In comparison, the number of IDEGs in the Mu_E vs. Mu_A and Mu_C vs. Mu_D stages is relatively small, with only 84 and 17 genes, respectively. This suggests that during the transitional periods at the beginning and termination of molting, regulatory processes in the muscle are accomplished through fine-tuning. These stage-specific expression dynamics reveal a hierarchical regulatory network that coordinates the molting process, from physiological preparation to structural remodeling and finally to post-molting recovery.

Besides the Mu_C vs. Mu_D stages, the other three molting stages exhibit the involvement of distinct signaling pathways. Specifically, nine pathways are active in Mu_A vs. Mu_C, eight in Mu_D vs. Mu_E, and three in Mu_E vs. Mu_A. Three core pathways are shared across these transitions: the GPCR signaling, steroid hormone-mediated signaling, and smoothened signaling pathways. Among these, the GPCR signaling pathway consistently harbors the highest number of IDEGs in each stage, underscoring its pivotal role as the central signaling hub during molting. The smoothened signaling pathway is a core component of the Hedgehog (Hh) cascade. It is centered on the key gene *Smo*, encoding protein Smoothened. This pathway is defined as “The series of molecular signals generated as a consequence of activation of the transmembrane protein Smoothened” in the Gene Ontology database. In this work, mucin-5AC-like, SUN domain-containing ossification factor, and bone morphogenetic protein genes were annotated to the smoothened signaling pathway. It is hypothesized that the smoothened signaling pathway may have a relationship with shell development during molting of *E. sinensis*.

In both fruit flies (*Drosophila melanogaster)* and cotton bollworms (*Helicoverpa armigera*), GPCRs have been found to receive the 20E signal, rapidly alter calcium levels, induce protein phosphorylation, and regulate gene transcription related to molting development, as well as the insect metamorphosis process [36,37]. In this work, GPCRs represent the most prominent regulatory components across molting stages in the muscle of *E. sinensis*, with distinct expression profiles shaping their functional diversity. In the Mu_A vs. Mu_C stages, the GPCR signaling pathway includes 5 genes with GPCR activity and characteristic structure: 1 up-regulated gene (*moody*) and 4 down-regulated genes (*GRM7*, *FMRFaR*, *mth2*, *gpr161*). These genes likely coordinate the transition from post-molt muscle remodeling to inter-molt stabilization.

The gene *gpr161* was down-regulated in the Mu_A vs. Mu_C stages and up-regulated in the Mu_D vs. Mu_E stages. It has been found to play a central role in development by regulating the hedgehog signaling pathway in both mice and zebrafish [33,34]. Research on the structure of *gpr161* indicates that it can regulate signaling pathways by binding to sterols [38]. Therefore, it is hypothesized that *gpr161* might affect the molting process through potential interactions with the sterol hormone-mediated signaling pathway based on its highest activity during the molt and post-molt periods.

The gene *FMRFaR* was also down-regulated in the Mu_A vs. Mu_C stages and up-regulated in the Mu_D vs. Mu_E stages. It is a receptor for FMRFamide. *FMRFaR* was found to mediate the intracellular calcium signaling, thereby playing an important role in maintaining neuronal excitability and regulating flight duration in fruit flies (*D. melanogaster*) [39]. In addition, *FMRFaR* can influence the locomotor behavior of the myrid bug (*Apolygus lucorum*) through energy metabolism pathways and motor protein-related pathways [40]. It is therefore hypothesized that in *E. sinensis*, *FMRFaR* may affect the muscle movement during molting by modulating neural signaling, metabolic pathways, and motor proteins. It exerts the greatest activity in the molt and post-molt periods.

The gene *mth2* was found to be down-regulated in the Mu_A vs. Mu_C stages and up-regulated in the Mu_E vs. Mu_A stages. It has been found to affect the lifespan regulation and oxidative stress resistance in fruit flies (*D. melanogaster*) [35]. In addition, it is a possible genetic marker for normal pigmentation development in shrimp [41]. It exerts the greatest activity in the post-molt period in *E. sinensis*.

Besides the genes with differential expression across stages, there are two unique IDEGs in the Mu_A vs. Mu_C stages (*GRM7* and *moody*). *GRM7* encodes the metabotropic glutamate receptor 7, which is essential for modulating neurotransmission in humans [42]. A mutant of *GRM7* may induce neurodevelopmental disorder. *moody* was found to mediate a signaling pathway in glial cells and regulate the nervous system insulation and influence sleep. Therefore, it is hypothesized that *GRM7* and *moody* may regulate the termination of molting by influencing the nervous system and neurotransmission in muscle.

There are three IDEGs unique in the Mu_D vs. Mu_E stages (*Kpna6*, *ADRB2*, *SSTR2*), which were verified to be up-regulated. *Kpna6* encodes a karyopherin alpha protein that interacts with the Keap1 protein, promotes the nuclear import of Keap1, and attenuates Nrf2 signaling. It plays a crucial role in regulating the Nrf2-dependent antioxidant response and maintaining cellular redox homeostasis [43]. *ADRB2* encodes the beta-2 adrenergic receptor. *SSTR2* encodes the somatostatin receptor 2. Both *ADRB2* and *SSTR2* are associated with signaling pathways and participate in cellular regulation through signal modulation. They have been widely studied in the treatment of diseases such as mammalian cancers and cardiomyopathy [44,45,46]. The differential expression of these genes suggests that the transition from the pre-molt to molt stages in *E. sinensis* may be related to signaling regulation mediated by nuclear transport, adrenergic receptors and somatostatin receptors.

Notably, the 7-TMH structure of these GPCRs and their function as the signaling receptor involved in cellular regulation are conserved across *Drosophila*, vertebrates, and *E. sinensis*. However, their specific contributions to crustacean molting remain speculative without direct functional validation, which needs to be further investigated. The differential engagement of GPCRs and their associated pathways across molting stages underscores the complexity of discontinuous crustacean growth. Unlike linear developmental processes, molting requires precise temporal coordination of gene expression, and GPCRs emerge as critical nodes linking hormonal cues to cellular responses. The stage-specific expression patterns highlight the dynamic rewiring of GPCR-mediated signaling, tailored to the physiological demands of each stage. The muscle-specific regulation observed here provides a mechanistic bridge between endocrinology and tissue adaptation, a previously understudied interface in crustacean biology.

These findings not only deepen our understanding of *E. sinensis* molting but also offer a comparative framework for studying discontinuous growth in other crustaceans and arthropods. Future investigations could explore the upstream regulators of GPCRs (e.g., hormone donors) and downstream effectors (e.g., calcium signaling pathways), unraveling the hierarchical control of molting. Ultimately, decoding these signaling networks may inform aquaculture practices, addressing challenges like molting disorders and premature maturation, thereby enhancing the sustainability of crustacean farming.

Environmental factors are also important external signals regulating the molting cycle of *E. sinensis*. Cues such as temperature and food can form coordinated regulation with the molecular mechanisms identified in this study through hormone-mediated signaling pathways. An optimal temperature (e.g., 20 ± 1 °C in this study) can promote the synthesis of molting hormone 20E and activate the expression of core genes in the GPCR signaling pathway. In addition, sufficient nutrition provides the material basis for energy metabolism, structural reconstruction, and regulation of gene expression. Certain substances involved in the signaling pathways for regulation of gene expression need to be synthesized through the consumption of nutrients. For example, the synthesis of steroid hormones in steroid hormone-mediated signaling pathways requires the intake of cholesterol as a substrate, and the synthesis of the receptors in signaling pathways needs amino acids as the raw materials. The proper aquaculture environment in this study ensured the stability of molting stages and laid a foundation for analyzing the regulatory association between environment and molecular pathways. Future research can focus on exploring the response patterns of candidate genes under environmental stresses to improve the comprehensive understanding of molting regulation.

In addition, some limitations still exist in this study. Firstly, samples from the inter-molt (C), pre-molt (D), and post-molt (A) stages were derived from our previously published work [5,6], while only the molt (E) stage samples were newly released. It is good that these samples were taken from the same batch of crabs, so the consistency of their growth environment can be guaranteed. Secondly, the sample size of three crabs per stage is relatively small, which is a common constraint in crustacean molting studies. It is challenging to obtain a large number of synchronized molting-stage individuals because molting is asynchronous and juvenile crabs are sensitive to environmental stress. Third, functional validation of these GPCRs is lacking, which limits the confirmation of the direct causal roles of the identified GPCRs in molting regulation. Therefore, a wider and larger size of sample collection is needed in further studies, and 20E induction, GPCR silence/overexpression, and MIH silencing can be conducted to verify the functional validity of candidate GPCR genes and clarify their regulatory roles in the molting signaling pathway in future research.

## 5. Conclusions

Molting is a crucial physiological process that affects the growth and development of *E. sinensis*. Abnormalities in the molting process may significantly impact both the survival rate and quality of the crabs. The regulatory mechanisms vary across different stages of the molting cycle. In this work, ATAC-seq and RNA-seq were integrated to analyze the signaling pathways involved in adjacent molting stages. The results revealed that the GPCR signaling, steroid hormone-mediated signaling, and smoothened signaling pathways were all involved in regulating three molting stages, with the GPCR signaling pathway playing a dominant role across all three stages. Further structural analysis and RT-qPCR experiments validated 8 GPCRs participating in the molting process. Among them, *GRM7* and *moody* are specific to the Mu_A vs. Mu_C stages; *Kpna6*, *ADRB2*, and *SSTR2* are unique to the Mu_D vs. Mu_E stages; *FMRFaR* and *gpr161* function in both the Mu_A vs. Mu_C and Mu_D vs. Mu_E stages; and *mth2* was identified in both the Mu_A vs. Mu_C and Mu_E vs. Mu_A stages. These findings provide a set of candidate signaling genes and pathways that may be useful targets for future functional studies and, ultimately, for genetic improvement strategies.

## Figures and Tables

**Table 1:** Results of ATAC-seq analysis of *E. sinensis* muscle in the molt period.

Sample	Clean Reads	Clean Ratio	Q20	Q30	Mapped Reads ^1^	Peak	Summits
Mu_E_1	43,217,675	67.5%	93.8%	86.0%	36,107,288 (83.6%)	52,473	68,956
Mu_E_2	41,023,204	61.9%	95.3%	89.3%	24,335,132 (59.3%)	44,610	56,607
Mu_E_3	43,139,257	68.3%	94.2%	87.3%	35,556,488 (82.4%)	49,831	68,351

^1^ The number of reads mapped to non-mitochondrial regions (the percentage in parentheses represents the percentage of reads mapped to non-mitochondrial regions relative to clean reads).

**Table 2:** Results of RNA-seq analysis of *E. sinensis* muscle in the molt period.

Sample	Clean Reads	Clean Ratio	Q20	Q30	Mapped Reads ^1^
Mu_E_1	28,862,970	96.7%	96.3	91.4	23,908,337 (82.8%)
Mu_E_2	30,411,926	97.3%	96.3	91.5	25,329,334 (83.3%)
Mu_E_3	30,257,948	95.9%	96.4	91.6	24,617,362 (81.4%)

^1^ The number of reads mapped to non-mitochondrial regions (the percentage in parentheses represents the percentage of reads mapped to non-mitochondrial regions relative to clean reads).

**Table 3:** IDEGs in each molting stage based on the integrated analysis.

Molting Stage	IDEGs	Up-Regulated IDEGs	Down-Regulated IDEGs
Mu_C vs. Mu_D	17	12	5
Mu_D vs. Mu_E	491	327	164
Mu_E vs. Mu_A	84	60	24
Mu_A vs. Mu_C	491	40	451

**Table 4:** Structural predictions for candidate GPCRs across different molting stages.

Stage	Code	Gene ID	Gene Name	TMHMM ^1^	PSIPRED ^2^	SWISS-MODEL ^3^
Mu_A vs. Mu_C	U244	evm.TU.CM024127.1.244	*FMRFaR*	6	7	7
	U116	evm.TU.CM024146.1.116	*GRM7*	7	7	7× 2
	U176	evm.TU.CM024164.1.176	*gpr161*	7	7	7
	U314	evm.TU.CM024134.1.314	*mth2*	7	7	7
	U415	evm.TU.CM024100.1.415	*moody*	7	7	7
Mu_D vs. Mu_E	U176	evm.TU.CM024164.1.176	*gpr161*	7	7	7
	U63	evm.TU.CM024137.1.63	*Kpna6*	7	7	7
	U210	evm.TU.CM024165.1.210	*ADRB2*	8	7	7
	U244	evm.TU.CM024127.1.244	*FMRFaR*	6	7	7
	U288	evm.TU.CM024152.1.288	*SSTR2*	7	7	7
	U276	evm.TU.CM024144.1.276	*rhodopsin-like*	1	1	2
	U157	evm.TU.CM024104.1.157	*HTR4*	6	6	7
Mu_E vs. Mu_A	U314	evm.TU.CM024134.1.314	*mth2*	7	7	7
	U66	evm.TU.CM024136.1.65_evm.TU.CM024136.1.66	*sky*	0	2	7

^1^ number of TMHs predicted by TMHMM; ^2^ number of TMHs predicted by PSIPRED; ^3^ number of TMHs predicted by SWISS-MODEL.

**Table 5:** Expression of GPCRs in different molting stages.

Code	Gene ID	Gene Name	Stage	Log_2_FoldChange	Duplicated
U116	evm.TU.CM024146.1.116	*GRM7*	Mu_A vs. Mu_C	−7.62	NA
U415	evm.TU.CM024100.1.415	*moody*	Mu_A vs. Mu_C	1.97	NA
U63	evm.TU.CM024137.1.63	*Kpna6*	Mu_D vs. Mu_E	3.22	NA
U210	evm.TU.CM024165.1.210	*ADRB2*	Mu_D vs. Mu_E	4.53	NA
U288	evm.TU.CM024152.1.288	*SSTR2*	Mu_D vs. Mu_E	2.06	NA
U176	evm.TU.CM024164.1.176	*gpr161*	Mu_A vs. Mu_C	−1.78	duplicated in two stages
			Mu_D vs. Mu_E	2.07	
U244	evm.TU.CM024127.1.244	*FMRFaR*	Mu_A vs. Mu_C	−3.99	duplicated in two stages
			Mu_D vs. Mu_E	2.96	
U314	evm.TU.CM024134.1.314	*mth2*	Mu_A vs. Mu_C	−2.14	duplicated in two stages
			Mu_E vs. Mu_A	1.92	

## Data Availability

The data presented in this study are openly available in GEO database with the reference number GSE299867.

## Supplementary Materials

The following supporting information can be downloaded at https://www.mdpi.com/article/10.3390/biom16010108/s1, Supplementary Table S1. Primer sequences and efficiencies for candidate GPCR genes and housekeeping genes in RT-qPCR experiment. Supplementary Table S2. List of IDEGs. Supplementary Figure S1. Results of structure analysis with TMHMM for candidate GPCRs. Supplementary Figure S2. Results of structure analysis with PSIPRED for candidate GPCRs. Supplementary Figure S3. Results of structure analysis with SWISS-MODEL for candidate GPCRs.

## References

[1] Mykles D.L. (2021). Molting in crustaceans: A complex regulatory process. Front. Endocrinol..

[2] Kang X., Tian Z., Wu J., Mu S. (2012). Molt stages and digestive enzyme activity in *Eriocheir sinensis*. J. Fish. China.

[3] Chang E.S., Mykles D.L. (2011). Regulation of crustacean molting. Gen. Comp. Endocrinol..

[4] Yue W., Chen Y., Chen X., Hou X., Wang J., Wang C. (2021). Physiological and gene expression profiles of leg muscle provide insights into molting-dependent growth of Chinese mitten crab (*Eriocheir sinensis*). Reprod. Breed..

[5] Sun Z., Li J., Lv L., Gou Y., Wang B., Hao T. (2022). Integration of ATAC-seq and RNA-seq identifies active G-protein coupled receptors functioning in molting process in muscle of *Eriocheir sinensis*. Front. Mar. Sci..

[6] Wang B., Li J., Zhang M., Li A., Song Z., He Z., Sun J. (2025). Integration of ATAC-seq and RNA-seq reveals signal regulation during post-molt and inter-molt stages in muscle of *Eriocheir sinensis*. Front. Mar. Sci..

[7] Wang Y., Yang Z.G., SHEN C., Yao Q.Q., Zeng Q.T., Liu Q.B., Cheng Y.Q. (2014). The full length cDNA cloning and expression analysis of EcR from the Chinese mitten crab (*Eriocheir sinensis*). J. Fish. China.

[8] Wang Y., Yang Z.G., Guo Z.H., Yao Q.Q. (2013). The full length cDNA cloning and expression analysis of RXR from the Chinese mitten crab (*Eriocheir sinensis*). J. Fish. China.

[9] Yao Q.Q., Yang Z.G., Wang Y., Guo Z.H., Liu Q.B., Shi Q.Y., Cheng Y.X. (2015). Full length cDNA cloning of the chitinase gene (HXchit) and analysis of expression during the molting cycle of the Chinese mitten crab, *Eriocheir sinensis*. J. Fish. Sci..

[10] Wang W., Wu X.G., Xu L., Yao Q.Q., Cheng Y.X. (2015). Expression analysis of Myostatin during molting cycle in *Eriocheir sinensis*. J. Shanghai Ocean Univ..

[11] Hou X., Yang H., Chen X., Wang J., Wang C. (2021). RNA interference of mTOR gene delays molting process in *Eriocheir sinensis*. Comp. Biochem. Physiol. Part B Biochem. Mol. Biol..

[12] Huang S., Wang J., Yue W., Chen J., Gaughan S., Lu W., Lu G., Wang C. (2015). Transcriptomic variation of hepatopancreas reveals the energy metabolism and biological processes associated with molting in Chinese mitten crab, *Eriocheir sinensis*. Sci. Rep..

[13] Tian Z., Jiao C. (2019). Molt-dependent transcriptome analysis of claw muscles in Chinese mitten crab *Eriocheir sinensis*. Genes Genom..

[14] Li J., Sun J., Dong X., Geng X., Qiu G. (2019). Transcriptomic analysis of gills provides insights into the molecular basis of molting in Chinese mitten crab (*Eriocheir sinensis*). PeerJ.

[15] Buenrostro J.D., Wu B., Chang H.Y., Greenleaf W.J. (2015). ATAC-seq: A method for assaying chromatin accessibility genome-wide. Curr. Protoc. Mol. Biol..

[16] Liu G., Li X., Zhang Y., Wang X. (2023). Multi-omics integration identifies key regulatory networks during crustacean molting. Aquac. Res..

[17] Corces M.R., Trevino A.E., Hamilton E.G., Greenside P.G., Sinnott-Armstrong N.A., Vesuna S., Satpathy A.T., Rubin A.J., Montine K.S., Wu B. (2017). An improved ATAC-seq protocol reduces background and enables interrogation of frozen tissues. Nat. Methods.

[18] Jiang H., Lei R., Ding S.W., Zhu S. (2014). Skewer: A fast and accurate adapter trimmer for next-generation sequencing paired-end reads. BMC Bioinf.

[19] Li H., Durbin R. (2009). Fast and accurate short read alignment with Burrows-Wheeler transform. Bioinformatics.

[20] Ramirez F., Dundar F., Diehl S., Gruning B.A., Manke T. (2014). deepTools: A flexible platform for exploring deep-sequencing data. Nucleic Acids Res..

[21] Zhang Y., Liu T., Meyer C.A., Eeckhoute J., Johnson D.S., Bernstein B.E., Nusbaum C., Myers R.M., Brown M., Li W. (2008). Model-based analysis of ChIP-seq (MACS). Genome Biol..

[22] Yu G., Wang L.G., He Q.Y. (2015). ChIPseeker: An R/Bioconductor package for ChIP peak annotation, comparison and visualization. Bioinformatics.

[23] Love M.I., Huber W., Anders S. (2014). Moderated estimation of fold change and dispersion for RNA-seq data with DESeq2. Genome Biol..

[24] Bolger A.M., Lohse M., Usadel B. (2014). Trimmomatic: A flexible trimmer for Illumina sequence data. Bioinformatics.

[25] Andrews S. (2020). FastQC: A quality control tool for high throughput sequence data. Soil.

[26] Kim D., Langmead B., Salzberg S.L. (2015). HISAT: A fast spliced aligner with low memory requirements. Nat. Methods.

[27] Liao Y., Smyth G.K., Shi W. (2014). featureCounts: An efficient general purpose program for assigning sequence reads to genomic features. Bioinformatics.

[28] Young M.D., Wakefield M.J., Smyth G.K., Oshlack A. (2010). Gene ontology analysis for RNA-seq: Accounting for selection bias. Genome Biol..

[29] Krogh A., Larsson B., von Heijne G., Sonnhammer E.L.L. (2001). Predicting transmembrane protein topology with a hidden Markov model: Application to complete genomes. J. Mol. Biol..

[30] Hennig M., Bermel W., Spencer A., Dobson C.M., Smith L.J., Schwalbe H. (1999). Side-chain conformations in an unfolded protein: χ1 distributions in denatured hen lysozyme determined by heteronuclear 13C, 15N NMR spectroscopy. J. Mol. Biol..

[31] Waterhouse A., Bertoni M., Bienert S., Studer G., Tauriello G., Gumienny R., Heer F.T., de Beer T.A.P., Rempfer C., Bordoli L. (2018). SWISS-MODEL: Homology modelling of protein structures and complexes. Nucleic Acids Res..

[32] Akoglu H. (2018). User’s guide to correlation coefficients. Turk. J. Emerg. Med..

[33] Tschaikner P.M., Regele D., Rock R., Salvenmoser W., Meyer D., Bouvier M., Geley S., Stefan E., Aanstad P. (2021). Feedback control of the Gpr161-G(alphas)-PKA axis contributes to basal Hedgehog repression in zebrafish. Development.

[34] Hwang S.H., Somatilaka B.N., White K., Mukhopadhyay S. (2021). Ciliary and extraciliary Gpr161 pools repress hedgehog signaling in a tissue-specific manner. eLife.

[35] Song W., Ranjan R., Dawson-Scully K., Bronk P., Marin L., Seroude L., Lin Y.-J., Nie Z., Atwood H.L., Benzer S. (2002). Presynaptic regulation by the GPCR methuselah in Drosophila. Neuron.

[36] Zhao X.F. (2020). G protein-coupled receptors function as cell membrane receptors for the steroid hormone 20-hydroxyecdysone. Cell Commun. Signal..

[37] Wang D., Zhao W.L., Cai M.J., Wang J.X., Zhao X.F. (2015). G-protein-coupled receptor controls steroid hormone signaling in cell membrane. Sci. Rep..

[38] Hoppe N., Harrison S., Hwang S.H., Chen Z., Karelina M., Deshpande I., Suomivuori C.M., Palicharla V.R., Berry S.P., Tschaikner P. (2024). GPR161 structure uncovers the redundant role of sterol-regulated ciliary cAMP signaling in the Hedgehog pathway. Nat. Struct. Mol. Biol..

[39] Ravi P., Trivedi D., Hasan G. (2018). FMRFa receptor stimulated Ca2+ signals alter the activity of flight modulating central dopaminergic neurons in Drosophila melanogaster. PLoS Genet..

[40] Gao H., Tian Y., Zhang H., Li Y., Li C., Li B. (2024). Species-specific duplicated FMRFaR-like gene A62 regulates spontaneous locomotion in Apolygus lucorum. Pest Manag. Sci..

[41] Huang C.W., Chu P.Y., Wu Y.F., Chan W.R., Wang Y.H. (2020). Identification of functional SSR markers in freshwater ornamental shrimps Neocaridina denticulata using transcriptome sequencing. Mar. Biotechnol..

[42] Freitas G.A., Niswender C.M. (2023). GRM7 gene mutations and consequences for neurodevelopment. Pharmacol. Biochem. Behav..

[43] Sun Z., Wu T., Zhao F., Lau A., Birch C.M., Zhang D.D. (2011). KPNA6 (Importin alpha7)-mediated nuclear import of Keap1 represses the Nrf2-dependent antioxidant response. Mol. Cell. Biol..

[44] Wu F.Q., Fang T., Yu L.X., Lv G.S., Lv H.W., Liang D., Li T., Wang C.Z., Tan Y.X., Ding J. (2016). ADRB2 signaling promotes HCC progression and sorafenib resistance by inhibiting autophagic degradation of HIF1alpha. J. Hepatol..

[45] Kulik G. (2019). ADRB2-Targeting Therapies for Prostate Cancer. Cancers.

[46] Duan F., Li L., Liu S., Tao J., Gu Y., Li H., Yi X., Gong J., You D., Feng Z. (2024). Cortistatin protects against septic cardiomyopathy by inhibiting cardiomyocyte pyroptosis through the SSTR2-AMPK-NLRP3 pathway. Int. Immunopharmacol..

