# Supplementary figures and images for: Integrated Analysis of ATAC-Seq and RNA-Seq Reveals the Signal Transduction Regulation of the Molting Cycle in the Muscle of Chinese Mitten Crab (Eriocheir sinensis)
Source: Biomolecules. 2026 Jan 8;16(1):108. doi: 10.3390/biom16010108 (PMC12839205; doi:10.3390/biom16010108)

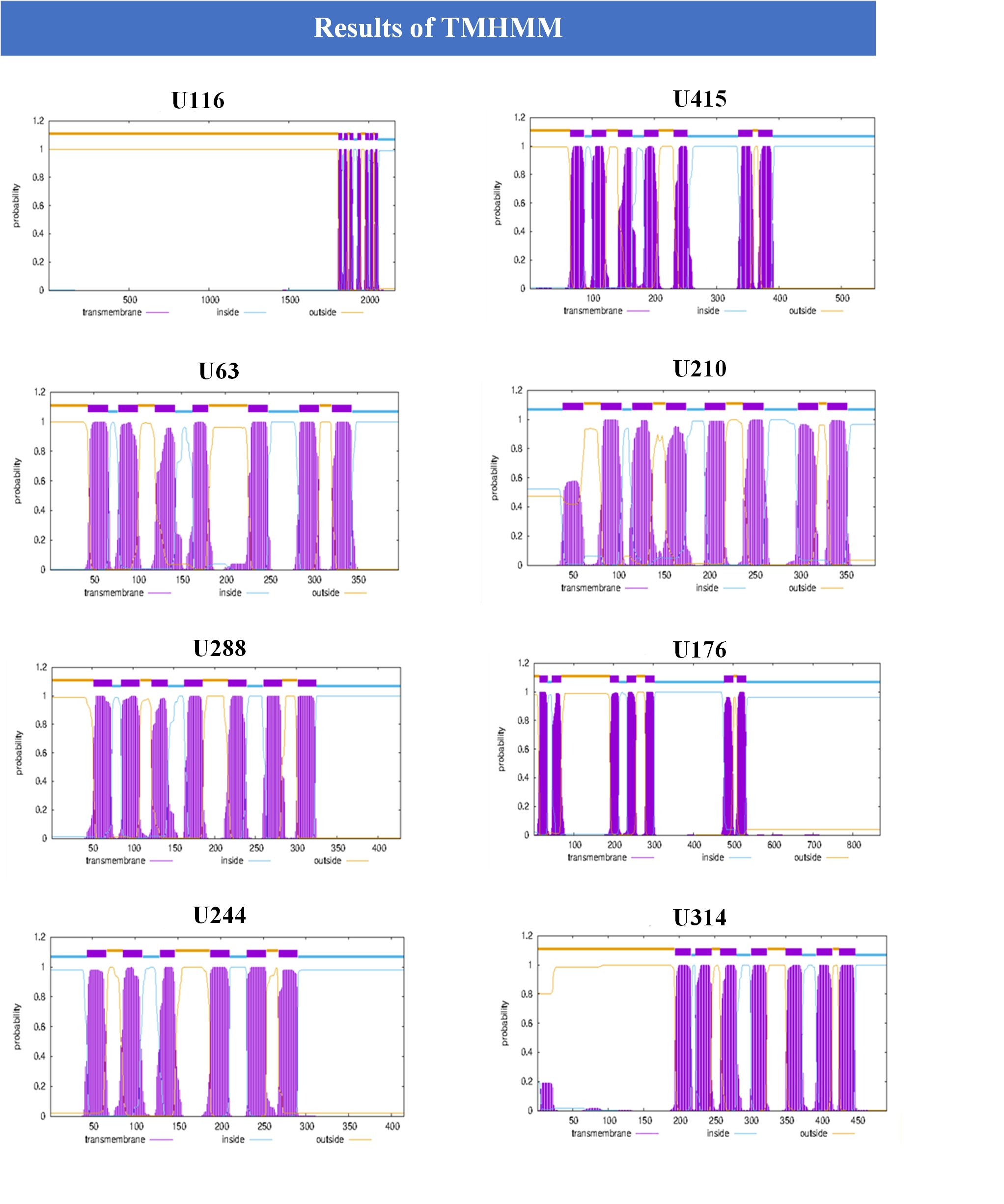

Supplement: Supplementary file 1 [file biomolecules-16-00108-s001.zip › Supplementary Figure S1.jpg]

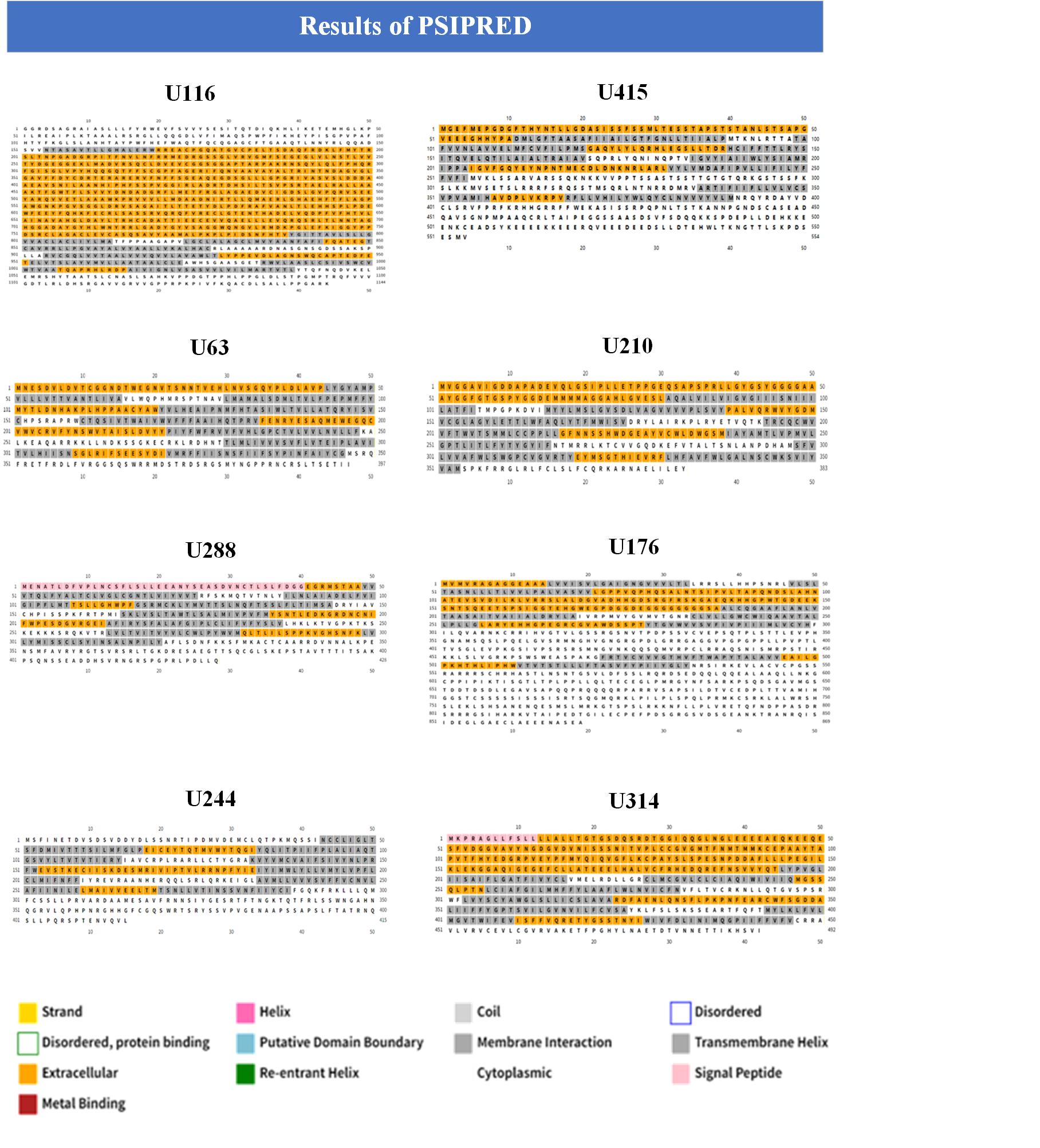

Supplement: Supplementary file 1 [file biomolecules-16-00108-s001.zip › Supplementary Figure S2.jpg]

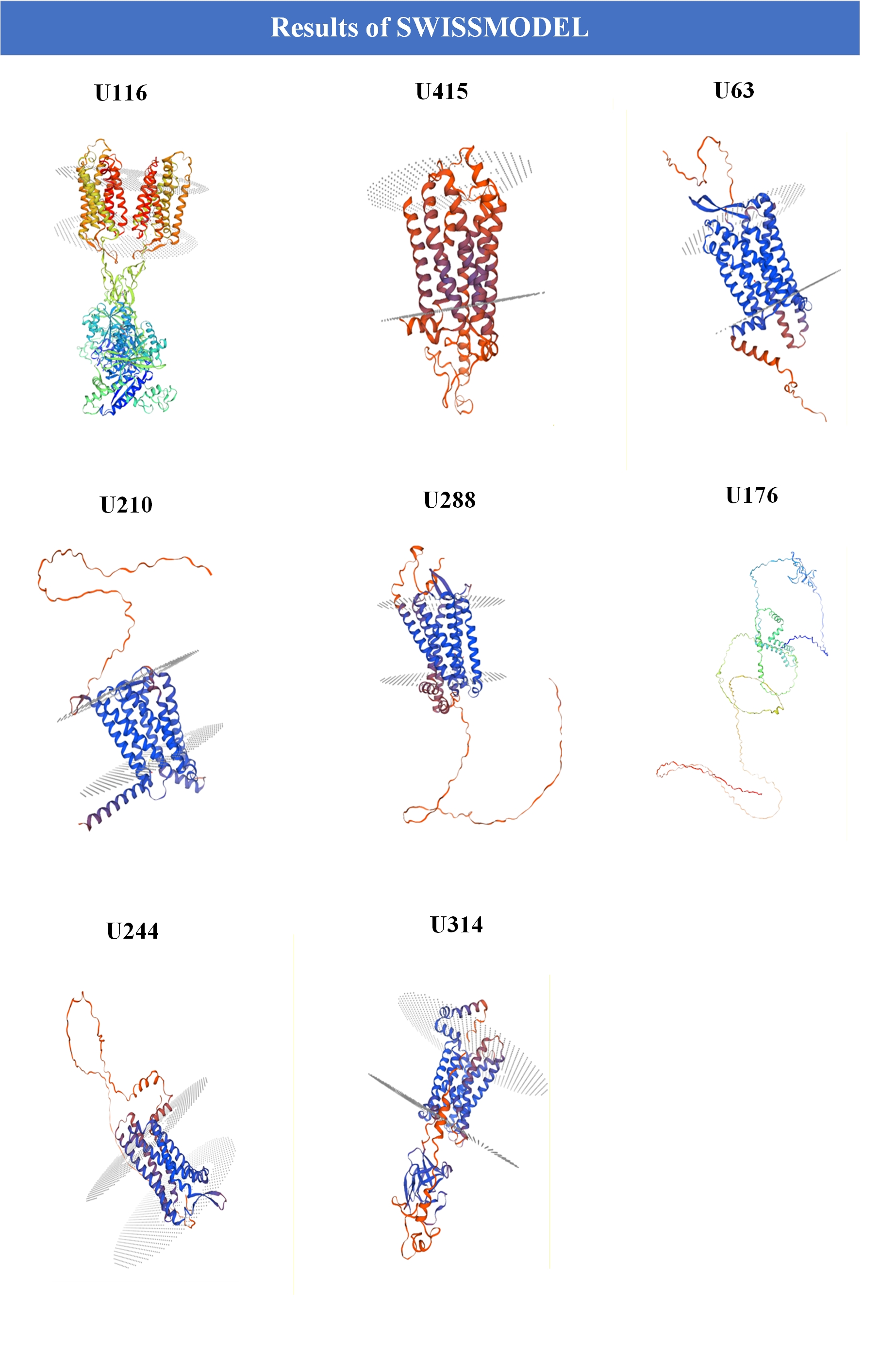

Supplement: Supplementary file 1 [file biomolecules-16-00108-s001.zip › Supplementary Figure S3.jpg]
